# An Ectopic Polycystic Kidney With Embryological and Clinical Significance: A Cadaveric Case Report

**DOI:** 10.7759/cureus.82868

**Published:** 2025-04-23

**Authors:** Juma Mwalimu, Kalpana Ramachandran, Vithya Thandayuthapani

**Affiliations:** 1 Anatomy, Sri Ramachandra Institute of Higher Education and Research, Chennai, IND

**Keywords:** cadaver case report, common iliac artery variant, embryology of kidney, pelvic kidney, polycystic kidney

## Abstract

Renal ectopia is a rare and usually asymptomatic anomaly. We report a cadaveric case of a 70-year-old male with the right pelvic polycystic kidney with two pairs of renal vessels and a curved right common iliac artery, observed during the routine dissection of the cadaver at a teaching institute. An ectopic kidney extended from the L4 to S1 vertebral levels. Its arterial supply consisted of a superior renal artery arising from the abdominal aorta just inferior to the origin of the inferior mesenteric artery, and an inferior renal artery originating from the aortic bifurcation. Similarly, venous drainage consisted of the superior renal vein, which drained into the inferior vena cava, and the inferior renal vein, which drained into the left common iliac vein. The renal pelvis was situated on the anteroinferior aspect of the kidney. Cross-sectional examination revealed obliteration of the normal renal parenchyma. This combination of findings has not been previously described in the literature. Although ectopic kidneys are often asymptomatic, recognizing the possibility of such unusual anatomical arrangements is crucial for accurate diagnosis and safer medico-surgical interventions when symptoms or related pathologies arise in the pelvic or retroperitoneal regions.

## Introduction

In adults, the kidneys are usually located between the 12th thoracic (T12) and third lumbar (L3) vertebrae. The excretory parts of the kidney develop from the metanephros, present in the lower lumbar and sacral region, while the collecting parts develop from the ureteric bud, which is a mesonephric diverticulum. The metanephric blastema fuses with the ureteric bud; if fusion does not take place, it will result in a congenital polycystic kidney. After the formation, the kidney ascends to the lumbar region during the sixth week; a failure to ascend will result in an ectopic kidney [1].

Renal arteries and renal veins supply blood to the kidney. The renal arteries are typically lateral branches of the abdominal aorta, arising at the level of the L2 vertebra, and the veins drain into the inferior vena cava. There is one renal artery on either side with its accompanying vein. Variations in renal arteries include lower level of origin, higher level of origin, accessory renal arteries, as well as pre-hilar division of renal arteries [2,3]. Abdominal aorta bifurcates at the level of L4 vertebra into right and left common iliac artery. The anatomy of the common iliac artery does not show variation in its origin, dimensions, course, and terminal division [4].

Congenital anomalies of the kidneys mostly remain undetected until adulthood and are sometimes an incidental finding during investigations. If a pelvic kidney is symptomatic, it presents with abdominal pain, which is associated with a mass felt in the lower abdominal region, only if it enlarges, recurrent urinary tract infections, and renal calculus formation [5]. These abnormalities need serious consideration with regard to surgical procedures involving the urinary system [6]. Therefore, congenital anomalies need to be highlighted because of their clinical implications.

## Case presentation

**Figure 1:**
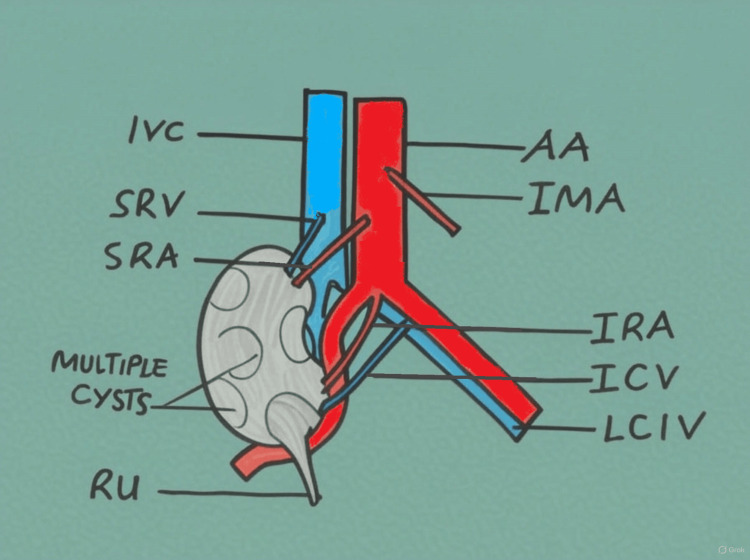
Schematic presentation of the case showing the right kidney with its vascular supply The right ureter extends from the lower pole of the kidney, descends anterior to the right common iliac artery, and then takes its normal course. Note the position of the kidney resting anterior to the common iliac artery, causing it to curve AA: abdominal aorta; IRA: inferior renal artery; IRV: inferior renal vein; IVC: inferior vena cava; LCIV: left common iliac vein; RU: right ureter; SRA: superior renal artery; SRV: superior renal vein Image credits: Juma Mwalimu (author)

During routine dissection of a formalin-fixed 70-year-old male cadaver at the Anatomy Department of a tertiary care medical college, a right pelvic polycystic kidney with two pairs of renal vessels and a curved course of the right common iliac artery was observed (Figure 1).

**Figure 2:**
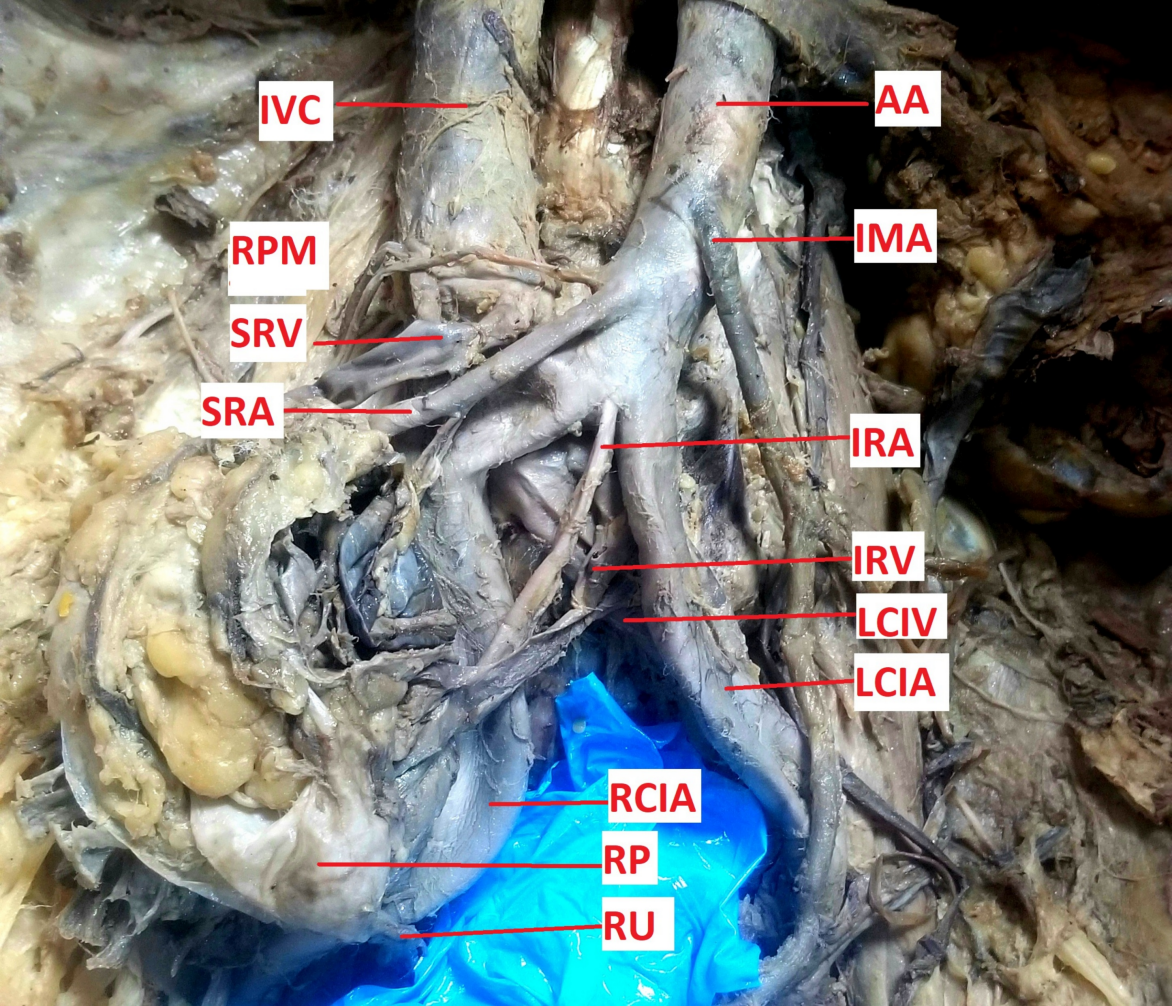
Image showing the position of the right pelvic polycystic kidney with its vascular variation The renal outline is poorly defined due to multiple distorted cysts AA: abdominal aorta; IRA: inferior renal artery; IRV: inferior renal vein; IVC: inferior vena cava; LCIV: left common iliac vein; RCIA: right common iliac artery; RP: renal pelvis; RPM: right psoas major; RU: right ureter; SRA: superior renal artery; SRV: superior renal vein

When the pelvis was dissected, the right kidney was located between L4 and S1 vertebrae, lying on the right psoas major and iliacus muscles. It had two renal arteries and two renal veins. The superior renal artery originated from the anterior surface of the abdominal aorta below the origin of the inferior mesenteric artery between L3 and L4 vertebrae; it was thicker than the inferior renal artery. It then coursed downwards and laterally to the upper medial border of the kidney to enter its hilum; along with it was the corresponding superior renal vein, which drained to the inferior vena cava. The inferior renal artery (accessory branch) originated from the anteroinferior aspect of bifurcation point of the abdominal aorta at the lower border of L4 vertebra, and then it coursed downwards and laterally to the lower medial border of the kidney; along with it was the corresponding inferior renal vein (accessory tributary), which drained to the left common iliac vein. The renal pelvis was directed more anteroinferiorly with the ureter situated on the lower pole (Figures 1, 2).

Coronal section of the right kidney showed poorly differentiated cortex and medulla. The cortex appeared to have multiple trabeculae with cysts, and in the medulla, there was loss of typical medullary pyramids, renal columns, as well as prominent enlarged renal calyces.

**Figure 3:**
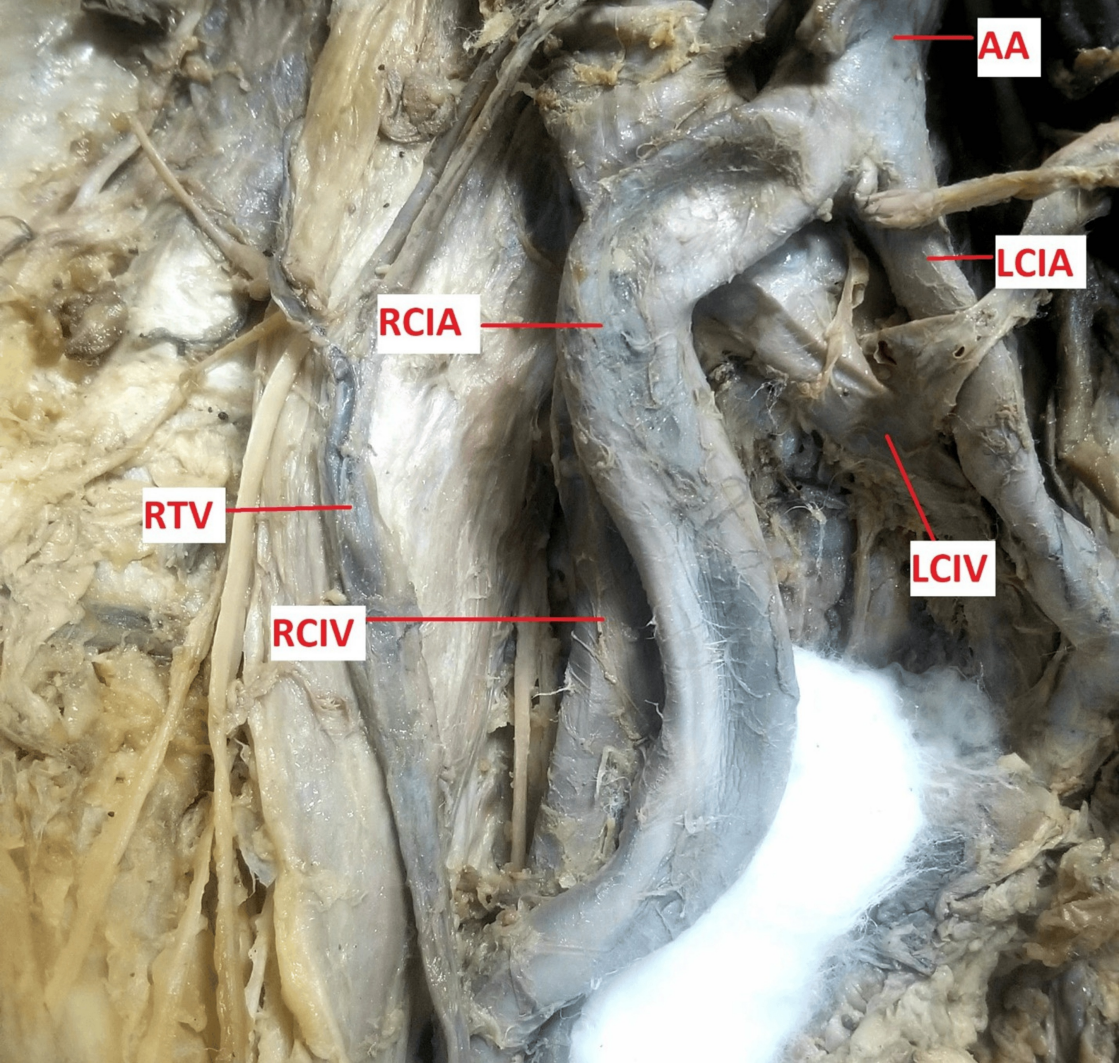
Image showing a curved right common iliac artery The kidney was reflected to the left side to expose the course of the right common iliac artery AA: abdominal aorta; LCIA: left common iliac artery; LCIV: left common iliac vein; RCIA: right common iliac artery; RCIV: right common iliac vein; RTV: right testicular vessels

The right common iliac artery was seen to make an S-shaped bend from its origin. It coursed obliquely downwards to the right, then curved medially on the pelvic brim, related to the psoas major on its posterior; it then divided into external and internal iliac arteries at the level of the sacroiliac joint. The curve occurred as a result of the medial border of the right kidney coming into contact with it. The right ureter arose from the pelvis on the lower pole of the kidney, coursed downwards medially anterior to the right common iliac artery, and then took a normal course in the true pelvis (Figure 3).

The left kidney was also polycystic, but was in a normal position in the lumbar region. The origin and course of renal vessels were normal except for the pre-hilar division of the left renal artery. The left renal pelvis was also in its normal location.

## Discussion

Kidney development is associated with multiple anomalies that vary in incidence: 7.07% lobulated kidneys, 5.05% polycystic kidneys, 5.05% hypoplastic or atrophic kidneys, 3.03% of the triplicate pelvis, 2.02% of pelvic kidneys, 2.02% of bifid pelvis, 1.01% renal agenesis, 1.01% of fused pelvic or pancake kidney, and 1.01% of malrotation kidney [5]. In this report, we discussed a polycystic pelvic kidney with its renal pelvis directed anteriorly. Ectopic kidneys clinically present with abdominal pain, hematuria, and calculus. Usually, an ectopic kidney is an incidental finding during investigations [6,7]. In our study, we had incidentally found an ectopic kidney during pelvic dissection, which had an unusual blood supply and an associated polycystic appearance of the kidney. However, the clinical history of our case could not be obtained.

Dogan et al. have reported a right ectopic pelvic kidney with two right renal arteries arising from the bifurcation of the abdominal aorta as anterior and posterior branches [8]. This is similar to our study, where the inferior renal artery arose from the abdominal aorta bifurcation. While both reports show two renal arteries, in the current study, the superior renal artery originated from the anterior aspect of the abdominal aorta below the inferior mesenteric artery at the level between the L3-L4 vertebrae. Common iliac artery variations are rare [4]. Kim et al. have reported a tortuous retro-psoas course of the right common iliac artery between the psoas and vertebral bodies. It did not bifurcate into internal and external iliac arteries but gave collateral branches that supplied the territories for external and internal iliac arteries [9]. In our study, an S-shaped right common iliac artery was observed, which later bifurcated normally into external and internal iliac arteries at the sacroiliac joint and then followed its normal course.

An ectopic kidney is secondary to the failure of the kidney to ascend from the pelvis to the lumbar region, where it is usually located during the sixth to ninth week of intrauterine life. The presence of a congenital polycystic kidney is due to the failure of fusion between the ureteric bud and metanephros. The ureteric bud will induce differentiation of the metanephros, which is influenced by transcription factors produced by the mesenchyme of the metanephric blastema, which also aids in epithelialization of the ureteric bud. Mutation of these factors can lead to multiple anomalies in the kidney [1].

Multiple variations in the abdominal aorta branching have been widely reported. Variations in the abdominal aorta branches play a crucial role in terms of surgeons' approach during abdominal, pelvic, as well as urological procedures [10]. We believe that these anatomical findings, as well as our observations, contribute to the existing literature on the topic.

## Conclusions

Ectopic and polycystic kidneys may occur as a single anomaly. Congenital anomalies of the kidney can be accompanied by unusual vascular supply to the kidney. Ectopic kidney can cause abnormal positioning/course of the viscera related to it, like curving of vessels. Clinicians should be mindful of the possibility of encountering such anatomical variants in clinical practice.
